# Author Correction: A weakly solvating electrolyte towards practical rechargeable aqueous zinc-ion batteries

**DOI:** 10.1038/s41467-024-52987-y

**Published:** 2024-11-15

**Authors:** Xin Shi, Jinhao Xie, Jin Wang, Shilei Xie, Zujin Yang, Xihong Lu

**Affiliations:** 1 MOE of the Key Laboratory of Bioinorganic and Synthetic Chemistry, The Key Lab of Low-carbon Chem & Energy Conservation of Guangdong Province, School of Chemistry, School of Chemical Engineering and Technology, Sun Yat-Sen University, Guangzhou, PR China; 2 School of Environment and Civil Engineering, Guangdong Engineering and Technology Research Center for Advanced Nanomaterials, Dongguan University of Technology, Dongguan, PR China

**Correction to:** *Nature Communications* 10.1038/s41467-023-44615-y, published online 05 January 2024

Correction text:

The original version of this article contained an error in Fig. 3e, where the authors mistakenly plotted the identical ex-situ XRD data for line A and B at different discharge states during the ex-situ characterization of NaV_3_O_8_·1.5H_2_O (NVO) in the weakly solvating electrolyte (WSE). As a result, Fig. 3f, the corresponding contour map of the ex-situ XRD patterns, was also incorrect, as it represents the same data as Fig. 3e. The correct line A are provided in the new Article file and the Source Data have been updated to reflect the correct data at the different discharge states. The figures 3e and 3f before and after correction are shown below. This correction should not impact the conclusions drawn in the work.

**Before Correction** 
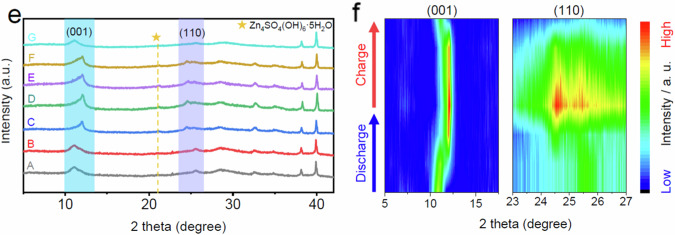


**After Correction**

**Figure:**
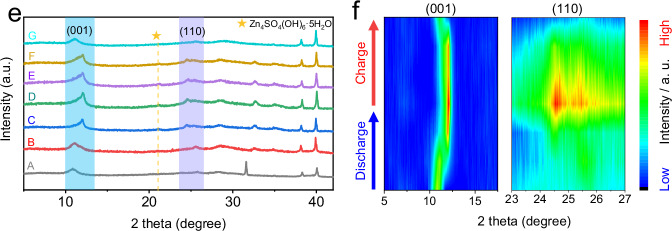
**Fig. 3e** ex-situ XRD patterns and **f** corresponding contour map.

The original version of this article contained another error in Supplementary Figs. 31a-b and Figs. 40a-b, where the authors mistakenly put the identical SEM images for different cycled electrodes. The correct SEM images of Supplementary Fig. 31a, b have been updated in the new Supplementary Information file to reflect the correct data for the surface morphology information of different cycled electrodes. Moreover, GCD curves of associated electrochemical data for Supplementary Figs. 31a and 40a have been added as Supplementary Figs. 31c and 40c. For reproducibility purposes, GCD curves of the newly assembled Zn/NVO and AA-Zn/NVO coin cell with WSE electrolyte have been provided in Supplementary Figs. 31d and 40d. In order to improve the clarity of the surface morphology for the electrodes, additional SEM images with different magnification are also provided in the new Supplementary Figs. 31e–h and 40e–h. The Supplementary Fig. 31 and Fig. 40 before and after correction are shown below. This correction should not impact the conclusions drawn in the work.

**Before Correction**

**Figure:**
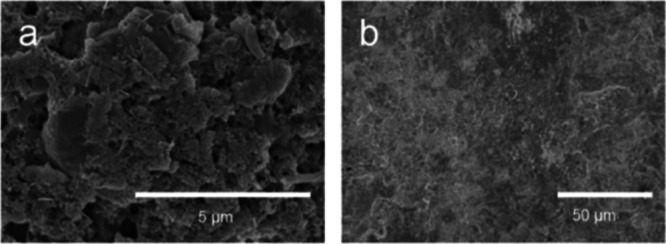
**Supplementary Fig. 31a, b** SEM images of NVO electrode after 50 cycles at 0.1 A g^−1^ in LCE and WSE.

**Figure:**
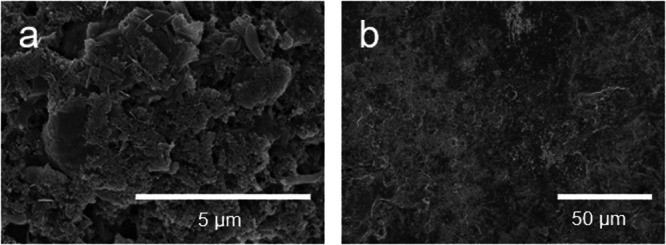
**Supplementary Fig. 40** SEM images of **a** NVO cathode and **b** Zn anode of AA-Zn/NVO after cycling stability test.

**After Correction**

**Figure:**
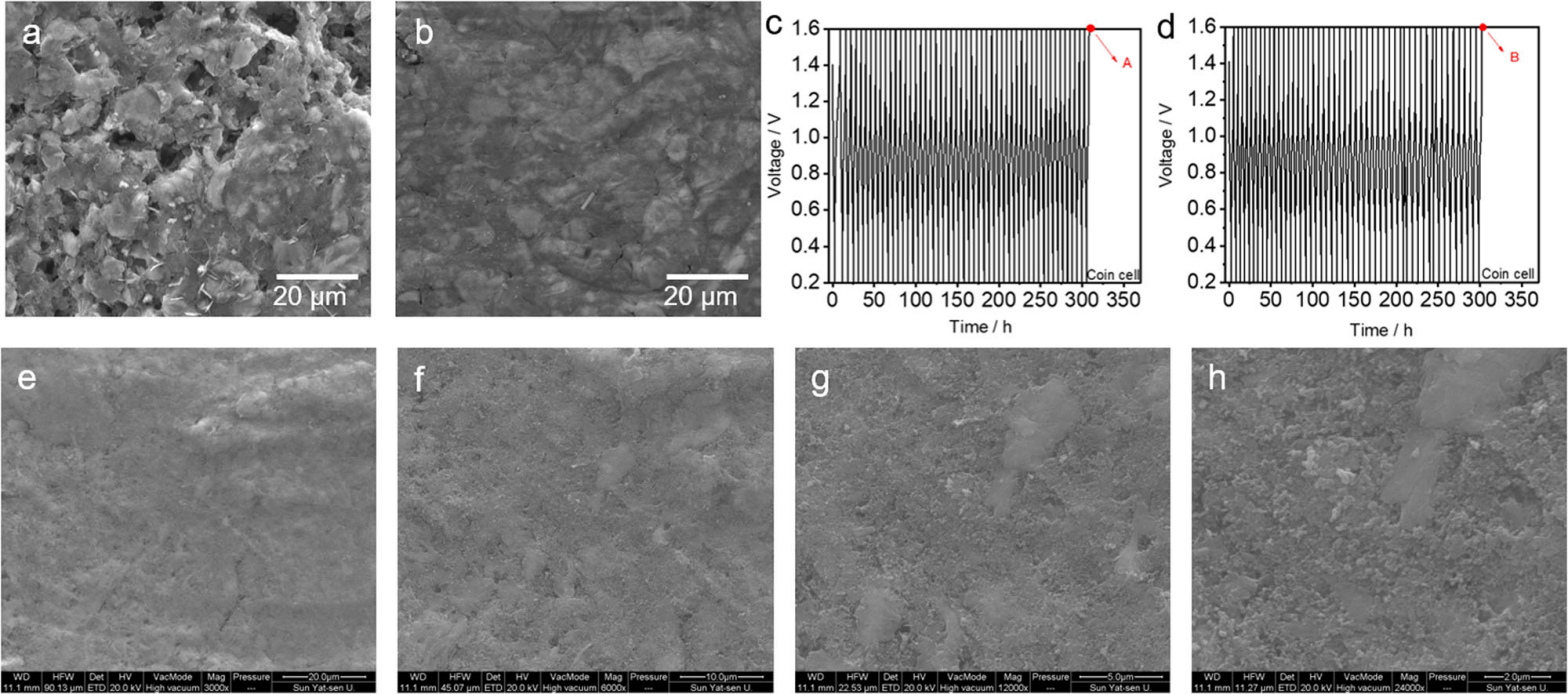
**Supplementary Fig. 31** SEM images of NVO electrode after 50 cycles at 0.1 A g^−1^ in **a** LCE and **b** WSE. **c** GCD curves of associated electrochemical data for Supplementary Fig. 31b. The tested voltage window for Zn/NVO coin cell with WSE is 0.2–1.6 V. The SEM image in Supplementary Fig. 31b was used to investigate the co-insertion phenomenon of H2O within NVO cathode in coin cell (charged state), which was recorded at point A in Supplementary Fig. 31c. **d** GCD curves of newly assembled Zn/NVO coin cell with WSE for reproducibility purposes. **e–h** SEM images from different magnifications of NVO electrode tested in newly assembled coin cell with WSE (charged state, recorded at point B in Supplementary Fig. 31d). The SEM images of NVO electrode tested in newly assembled coin cell with WSE indicates that the surface of NVO electrode characterized under different magnification is flat, in consistent with Supplementary Fig. 31b.

**Figure:**
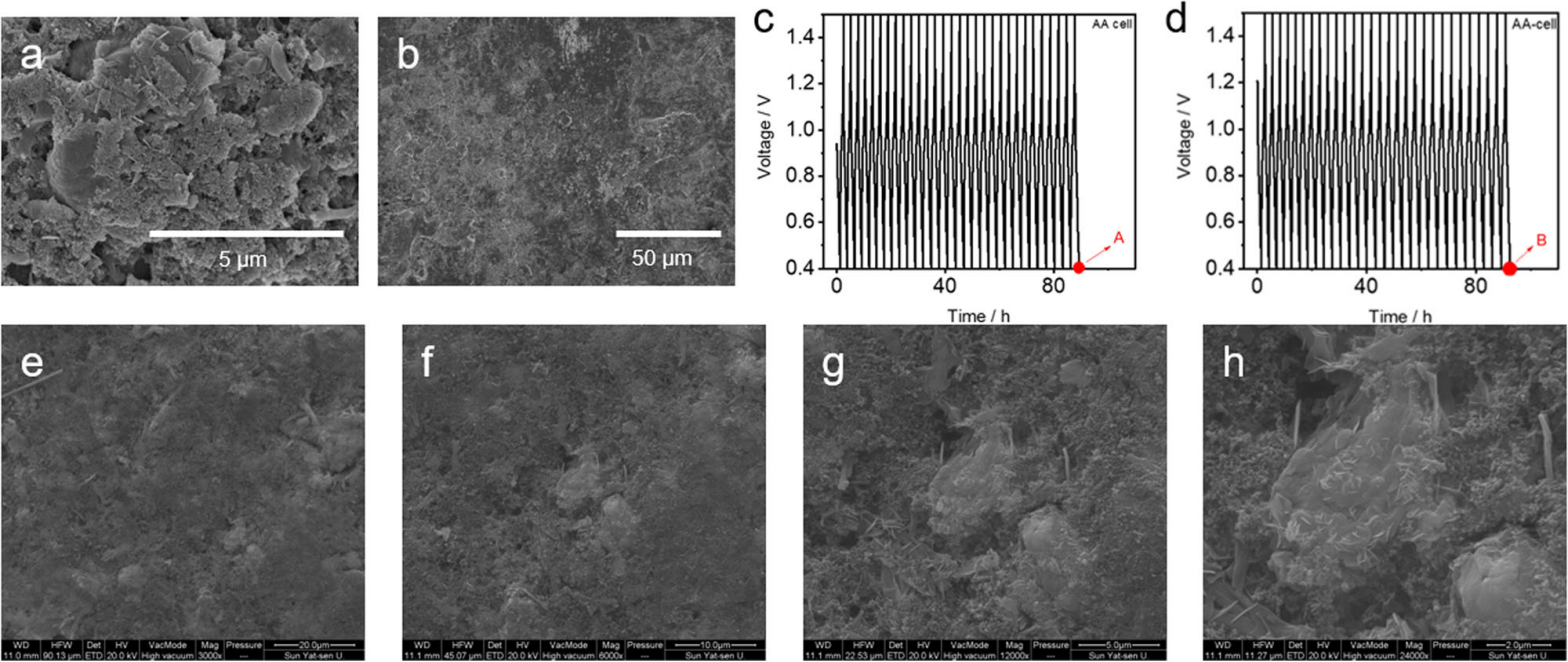
**Supplementary Fig. 40** SEM images of **a** NVO cathode and **b** Zn anode of AA-Zn/NVO with WSE after cycling stability test. **c** GCD curves of associated electrochemical data for Supplementary Fig. 40a. The tested voltage window for AA-Zn/NVO with WSE is 0.4**–**1.5 V. The current density is 0.66 C-rate. The SEM image in Supplementary Fig. 40a was used to investigate the morphology change of NVO after cycling test in AA-Zn/NVO with WSE (discharged state), which was recorded at point A in Supplementary Fig. 40c. **d** GCD curves of newly assembled AA-Zn/NVO with WSE for reproducibility purposes. **e–h** SEM images from different magnifications of NVO electrode tested in newly assembled AA-Zn/NVO with WSE (discharged state, recorded at point B in Supplementary Fig. 40d). The SEM images of NVO electrode tested in AA-Zn/NVO with WSE indicates that the morphology of NVO at low magnification is relatively homogeneous, while some clumps are observed at high magnification, which is also similar to the SEM images of Supplementary Fig. 40a.

## Supplementary information

Supplementary information updated.

## Source data

Updated source data.

