# Supplementary material for: A weakly solvating electrolyte towards practical rechargeable aqueous zinc-ion batteries
Source: Nat Commun. 2024 Jan 5;15:302. doi: 10.1038/s41467-023-44615-y (PMC10770389; doi:10.1038/s41467-023-44615-y)
Supplement: Supplementary file 1 — Supplementary Information [file 41467_2023_44615_MOESM1_ESM.pdf]

Supplementary Information

A weakly solvating electrolyte towards practical rechargeable aqueous zinc-ion batteries

Xin Shi,[1] Jinhao Xie,[1] Jin Wang,[1] Shilei Xie[2,*] Zujin Yang[1,*] and Xihong Lu[1,*]

[1] MOE of the Key Laboratory of Bioinorganic and Synthetic Chemistry, The Key Lab of Low-carbon Chem & Energy Conservation of Guangdong Province, School of Chemistry, School of Chemical Engineering and Technology, Sun Yat-Sen University, Guangzhou 510275, People's Republic of China

[2] School of Environment and Civil Engineering, Guangdong Engineering and Technology Research Center for Advanced Nanomaterials, Dongguan University of Technology, Dongguan 523808, People's Republic of China

*Corresponding author: xieshil@dgut.edu.cn, yangzj3@mail.sysu.edu.cn, luxh6@mail.sysu.edu.cn

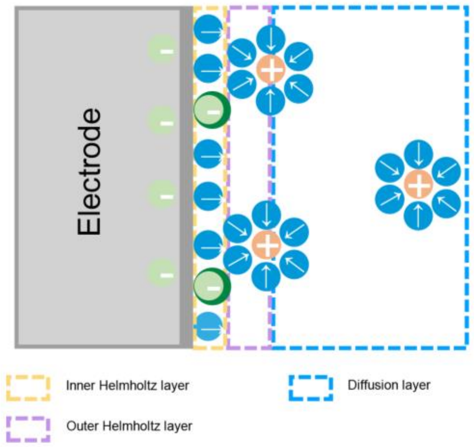

Supplementary Fig. 1 Schematics of Bockris-Devanathan-Müller model. The inner Helmholtz layer is consisted of solvent and anion while the solvated cation locates at outer Helmholtz layer.

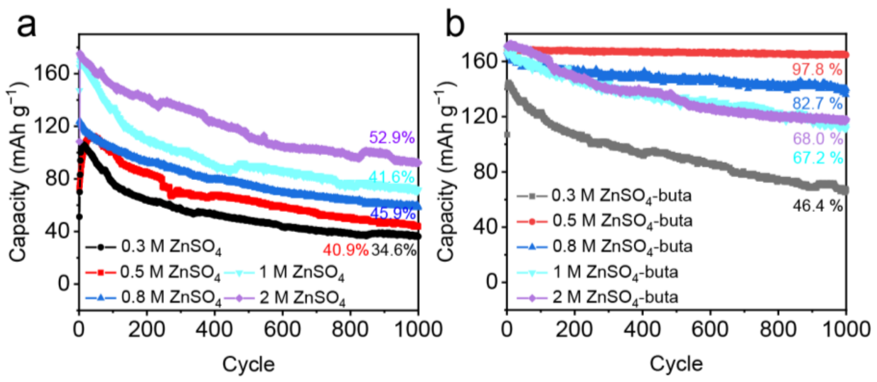

Supplementary Fig. 2 Cycling performance of Zn/NVO cells using **a** LCE and **b** WSE with different concentration.

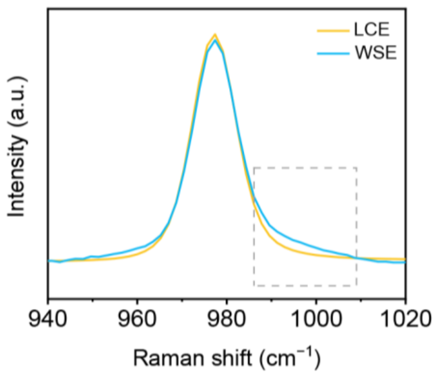

Supplementary Fig. 3 Overlapped Raman spectra of LCE and WSE.

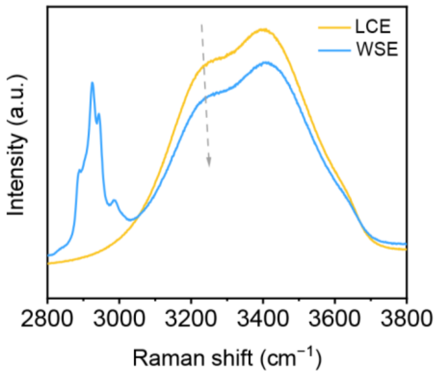

Supplementary Fig. 4 Raman spectra of LCE and WSE. The peaks at 2888 and 2941 cm^{-1} is corresponding to $-CH_3$ stretching vibration of butanone while the peaks at 2925 cm^{-1} is corresponding to $-CH_2-$ stretching vibration of butanone.

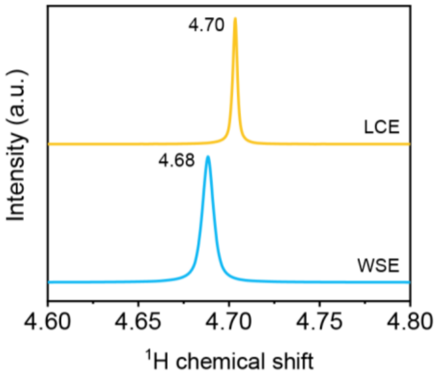

Supplementary Fig. 5 NMR spectra of LCE and WSE.

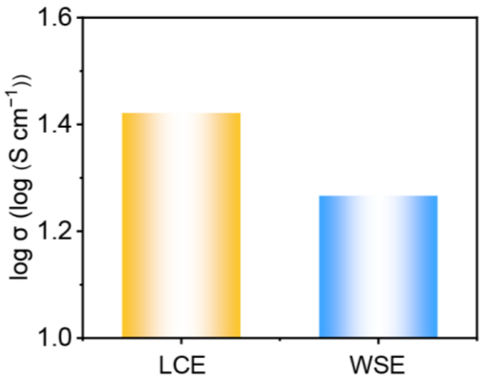

Supplementary Fig. 6 Ionic conductivity of LCE and WSE.

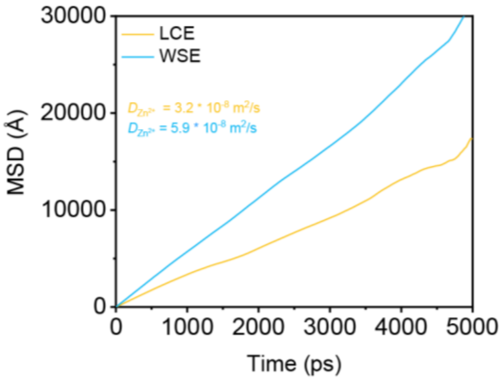

Supplementary Fig. 7 Calculated Zn^{2+} diffusion coefficient of LCE and WSE.

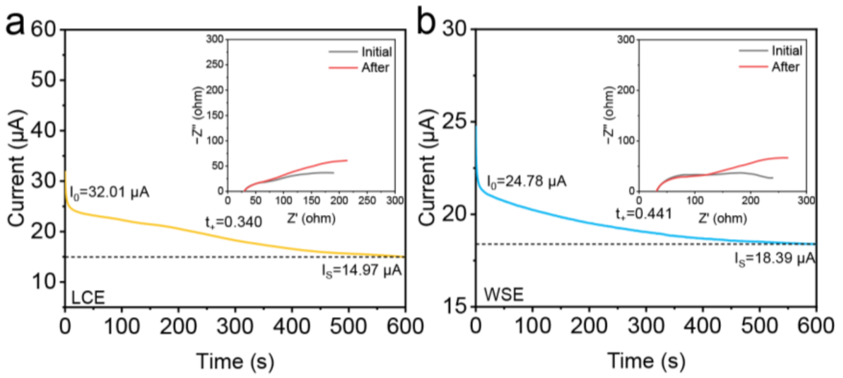

Supplementary Fig. 8 a and b I-t curves of LCE and WSE. Inset shows EIS spectra of Zn electrode before and following polarization.

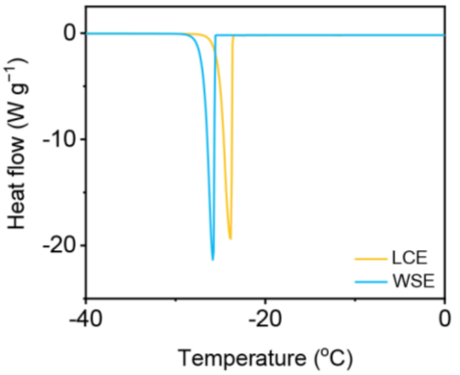

Supplementary Fig. 9 DSC curves for LCE and WSE under a cooling rate of 5 $^{o}C\ min^{-1}$.

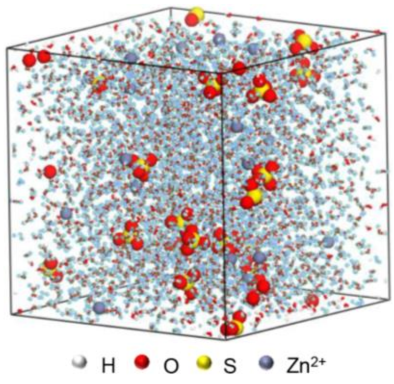

Supplementary Fig. 10 3D snapshot of LCE obtained from MD simulations.

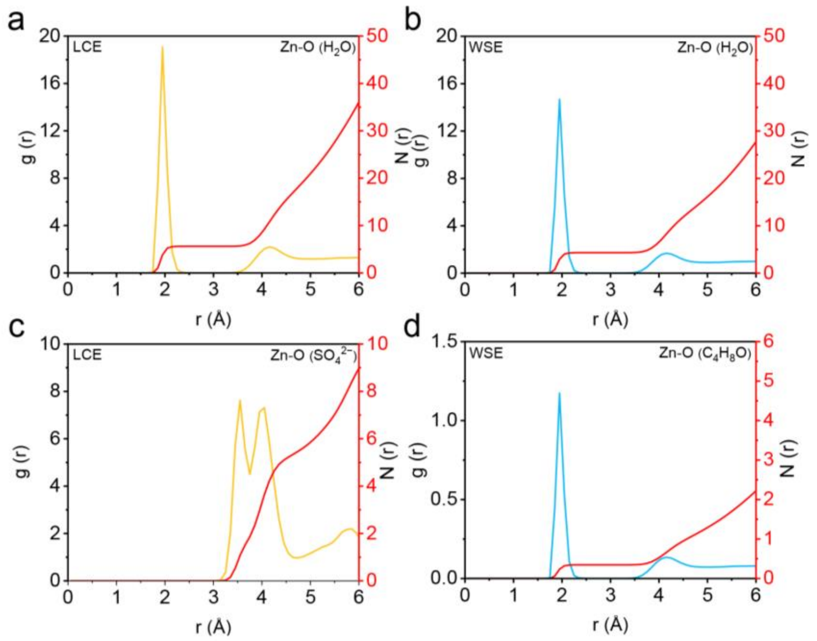

Supplementary Fig. 11 RDFs for **a** and **b** Zn^{2+}-O (H_2O), **c** Zn^{2+}-O (SO_4^{2-}) and **d** Zn^{2+}-O (C_4H_8O) collected from MD simulations in LCE and WSE.

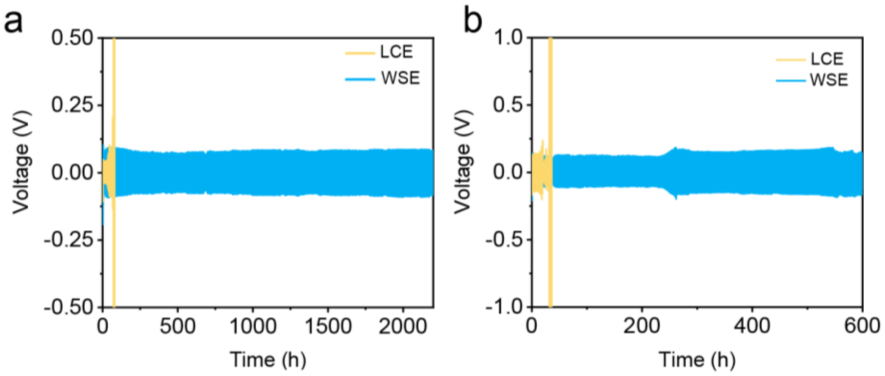

Supplementary Fig. 12 The voltage profiles of Zn/Zn symmetric cell tested in LCE and WSE at **a** 1 mA/mAh cm^{-2} and **b** 5 mA/5 mAh cm^{-2}.

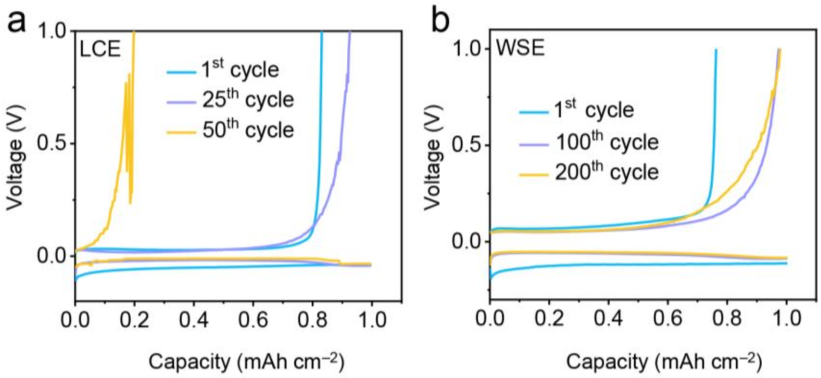

Supplementary Fig. 13 Plating/stripping curves of Ti/Zn cells in **a** LCE and **b** WSE.

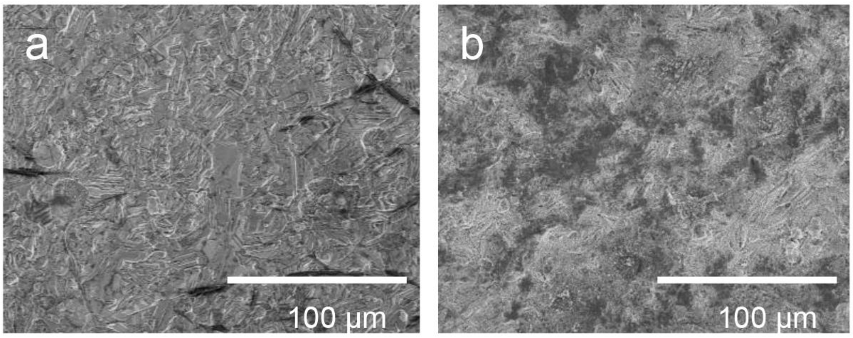

Supplementary Fig. 14 Morphology of Zn electrodes after 50 cycles in **a** LCE and **b** WSE.

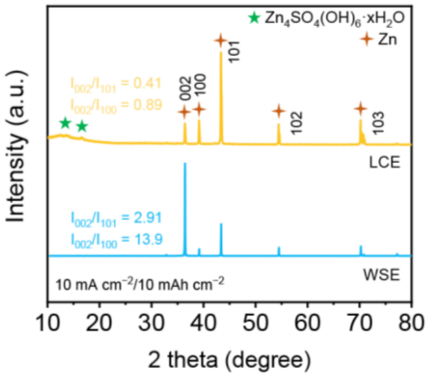

Supplementary Fig. 15 XRD pattern of Zn foil cycled after 50 cycles at 10 mA/10 mAh cm^{-2} in LCE and WSE.

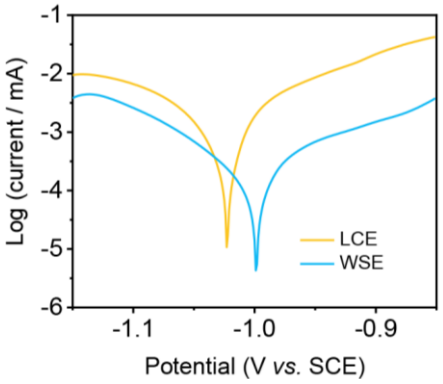

Supplementary Fig. 16 The Tafel plots of Zn plate tested in LCE and WSE at a scan rate of 1 mV s^{-1} using three-electrode system.

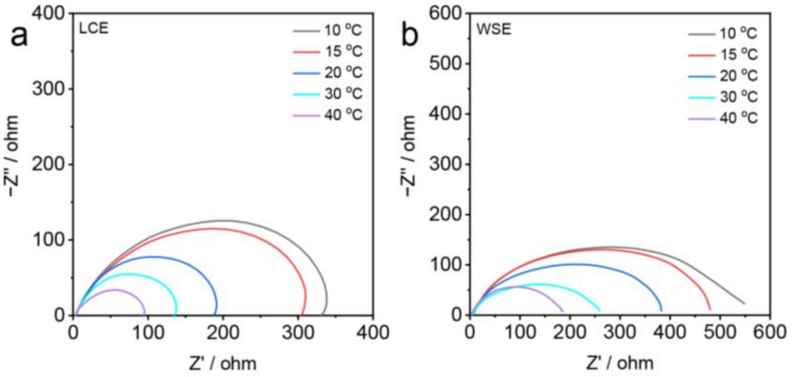

Supplementary Fig. 17 EIS spectra of Zn/Zn cells using **a** LCE and **b** WSE at different temperature.

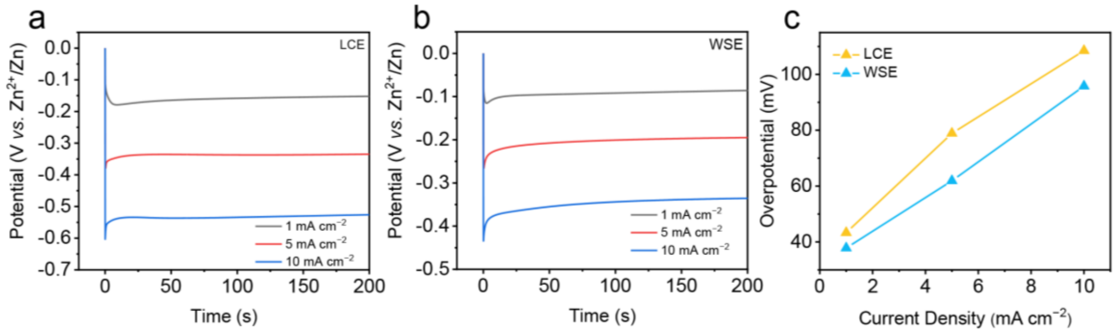

Supplementary Fig. 18 Nucleation overpotential of Zn electrode in different electrolytes.

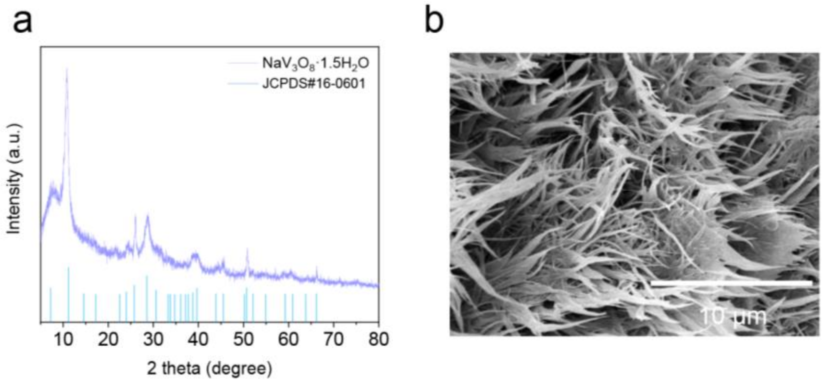

Supplementary Fig. 19 a XRD pattern and **b** SEM images of NVO.

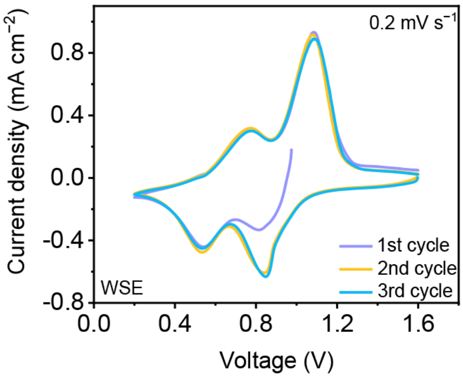

Supplementary Fig. 20 CV curves of NVO electrode at 0.2 mV s^{-1}.

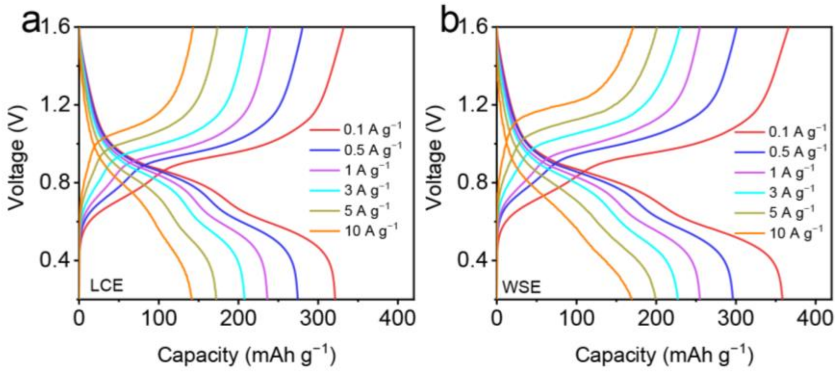

Supplementary Fig. 21 GCD curves of Zn/NVO cells using **a** LCE and **b** WSE.

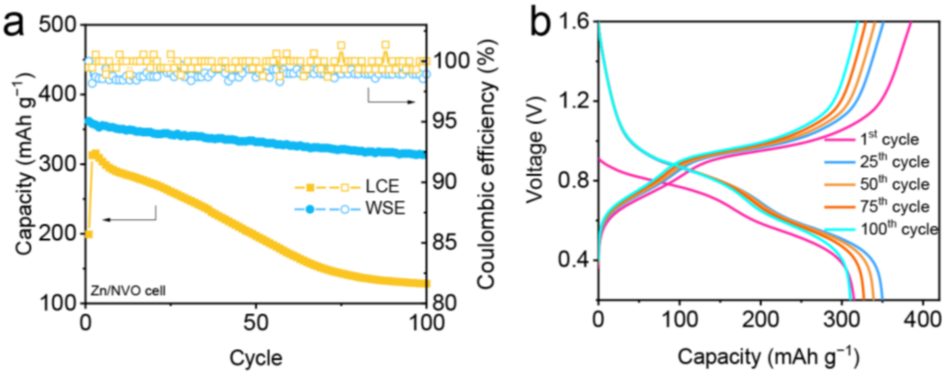

Supplementary Fig. 22 a Cycling stability of NVO cathode at 0.1 A g^{-1}. **b** Selected GCD curves of NVO cathode.

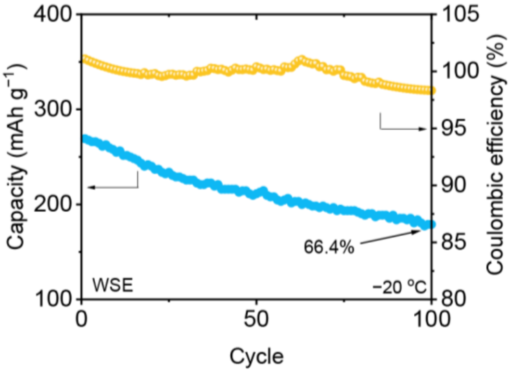

Supplementary Fig. 23 Low temperature cycling performance of Zn/NVO cell at 0.1 A g^{-1}.

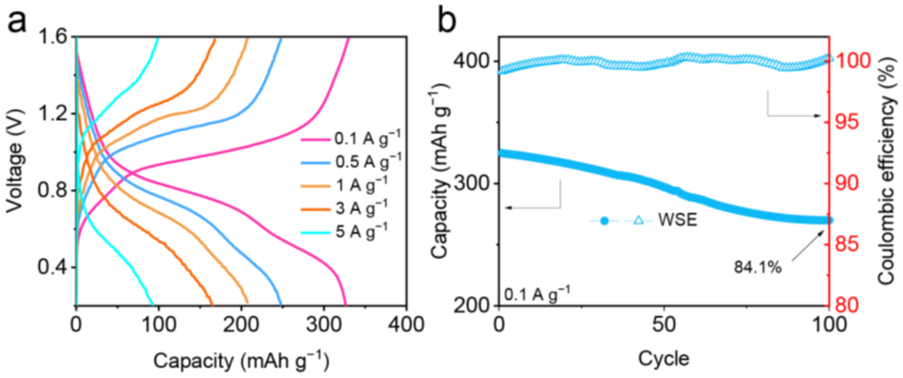

Supplementary Fig. 24 a GCD curves of Zn/h-NVO at different current densities. **b** Cycling stability of Zn/h-NVO at 0.1 A g^{-1}.

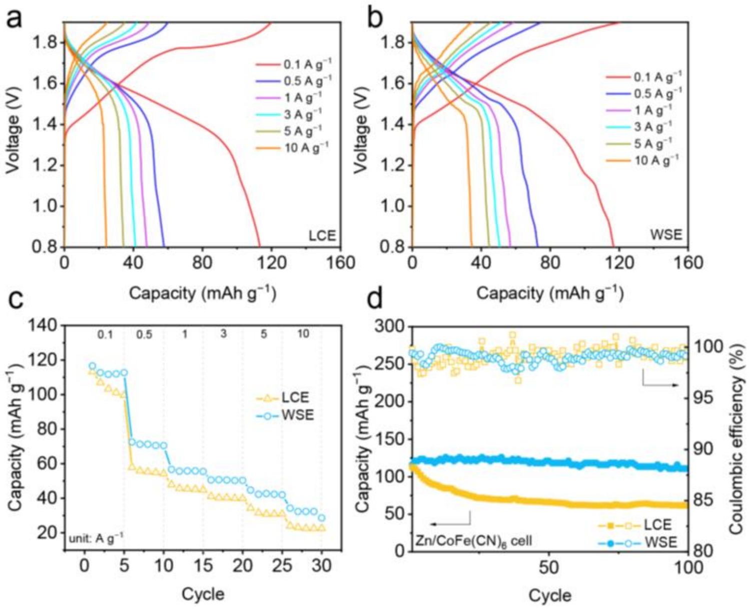

Supplementary Fig. 25 a, **b** GCD curves, **c** rate performance and **d** cycling stability of $CoFe(CN)_6$ cells using LCE and WSE.

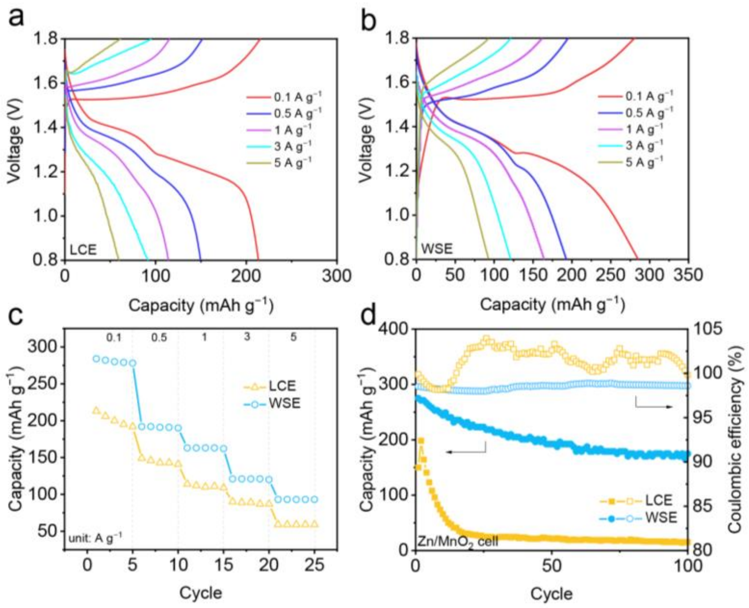

Supplementary Fig. 26 a, **b** GCD curves, **c** rate performance and **d** cycling stability of MnO_2 cells using LCE and WSE.

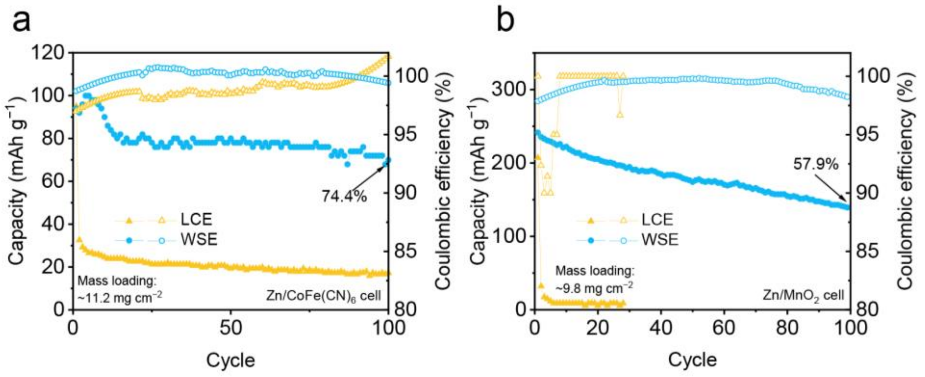

Supplementary Fig. 27 Cycling stability of high mass loading **a** Zn/$CoFe(CN)_6$ and **b** Zn/MnO_2 cells using LCE and WSE at 0.1 A g^{-1}.

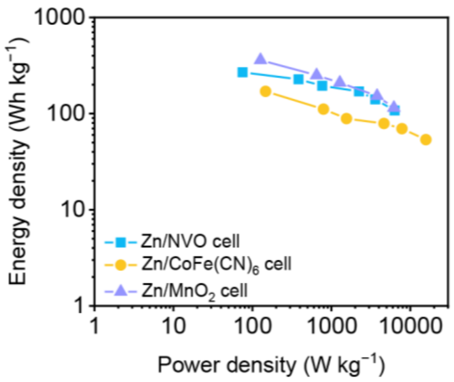

Supplementary Fig. 28 Ragone plots of Zn/NVO, Zn/$CoFe(CN)_6$ and Zn/MnO_2 cells using WSE.

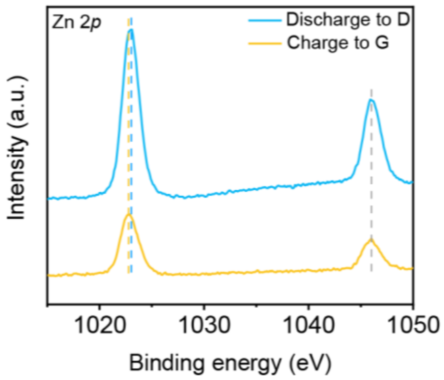

Supplementary Fig. 29 Zn 2*p* XPS spectra of NVO electrode at fully discharged and charged state in WSE.

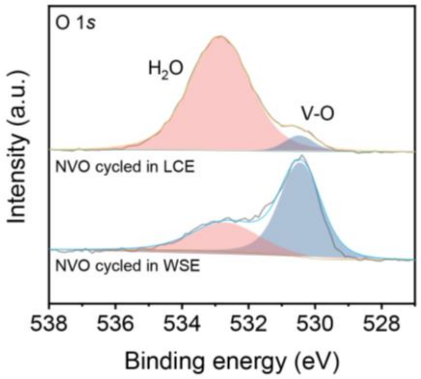

Supplementary Fig. 30 O 1*s* XPS spectra of NVO electrode after 50 cycles at 0.1 A g^{-1} in LCE and WSE.

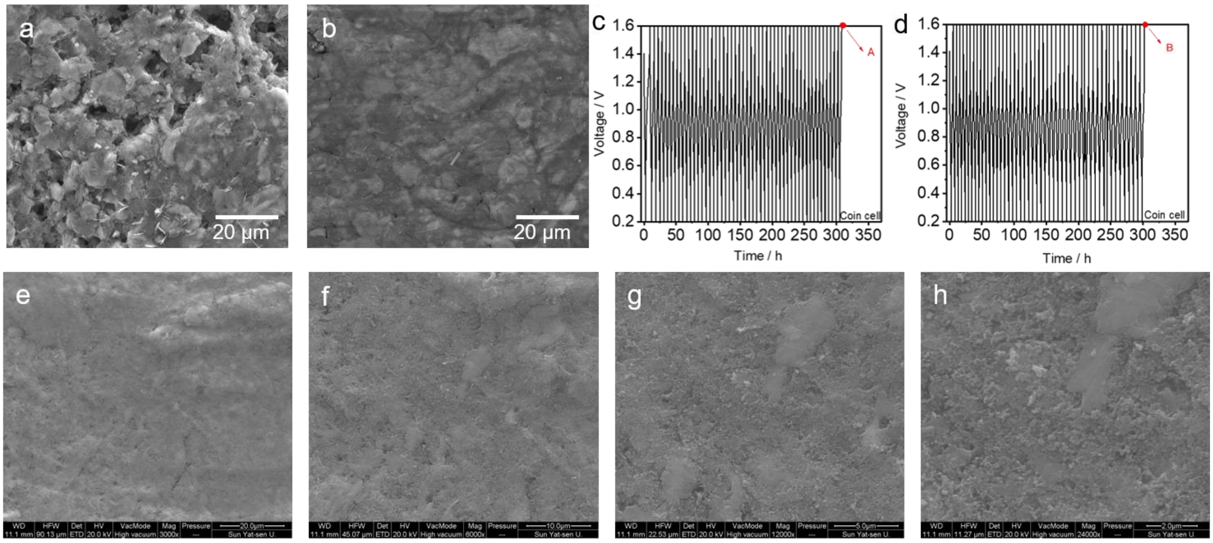

Supplementary Fig. 31 SEM images of NVO electrode after 50 cycles at 0.1 A g^{-1} in **a** LCE and **b** WSE. **c** GCD curves of associated electrochemical data for Supplementary Fig. 31b. The tested voltage window for Zn/NVO coin cell with WSE is 0.2-1.6 V. The SEM image in Supplementary Fig. 31b was used to investigate the co-insertion phenomenon of H_2O within NVO cathode in coin cell (charged state), which was recorded at point A in Supplementary Fig. 31c. **d** GCD curves of newly assembled Zn/NVO coin cell with WSE for reproducibility purposes. **e-h** SEM images from different magnifications of NVO electrode tested in newly assembled coin cell with WSE (charged state, recorded at point B in Supplementary Fig. 31d). The SEM images of NVO electrode tested in newly assembled coin cell with WSE indicates that the surface of NVO electrode characterized under different magnification is flat, in consistent with Supplementary Fig. 31b.

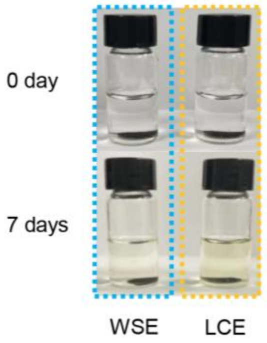

Supplementary Fig. 32 Optical images of NVO electrode immersed in LCE and WSE for 7 days.

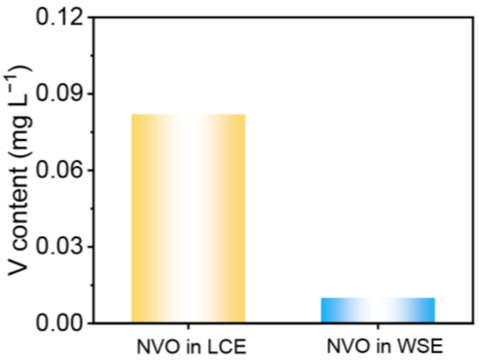

Supplementary Fig. 33 The dissolved V content after resting NVO in coin cells with LCE and WSE as electrolytes for 7 days.

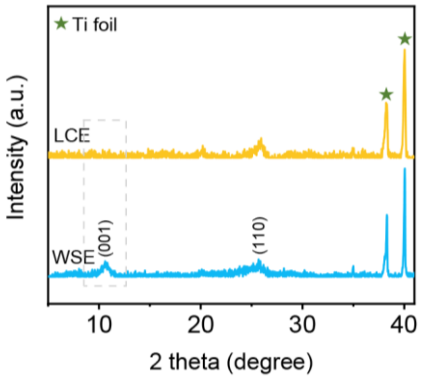

Supplementary Fig. 34 XRD patterns of NVO after soaking in LCE and WSE for 7 days.

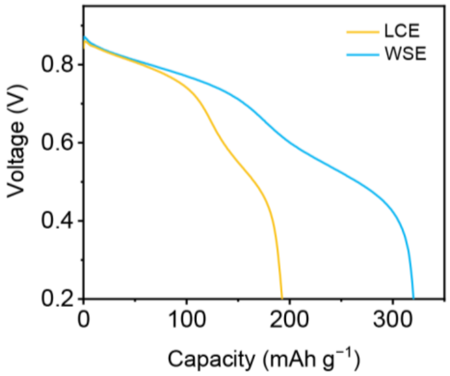

Supplementary Fig. 35 The first discharge curves of Zn/NVO cells after resting for 144 h in LCE and WSE.

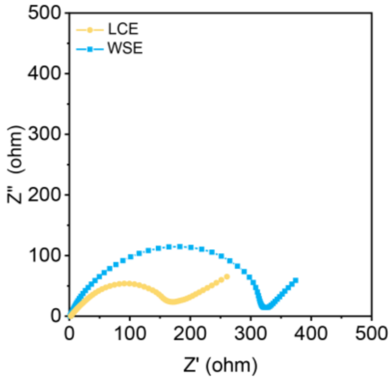

Supplementary Fig. 36 EIS spectra of Zn/NVO full cells based on LCE and WSE.

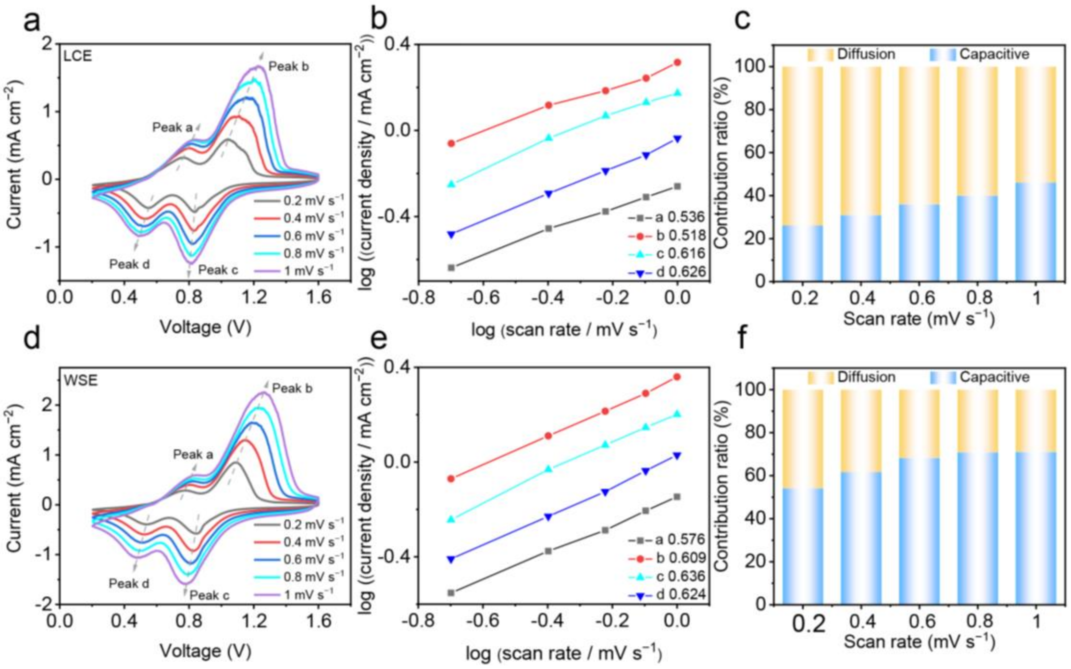

Supplementary Fig. 37 CV curves of Zn/NVO cells in **a** LCE and **d** WSE. Corresponding **b**, **e** *b* value and **c**, **f** capacitance contribution.

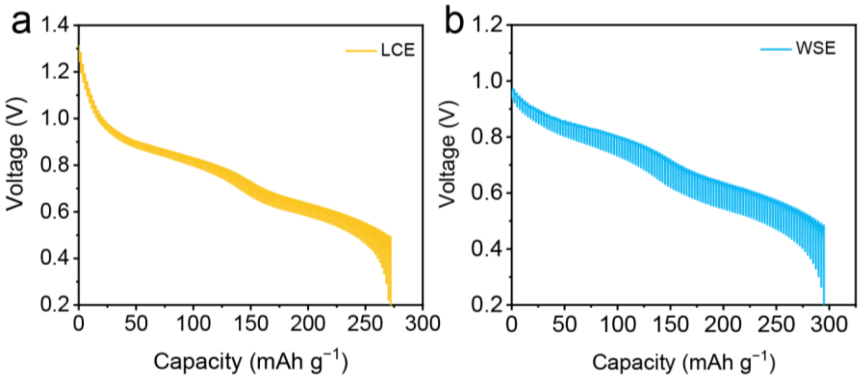

Supplementary Fig. 38 Discharge profiles of Zn/NVO cells in **a** LCE and **b** WSE.

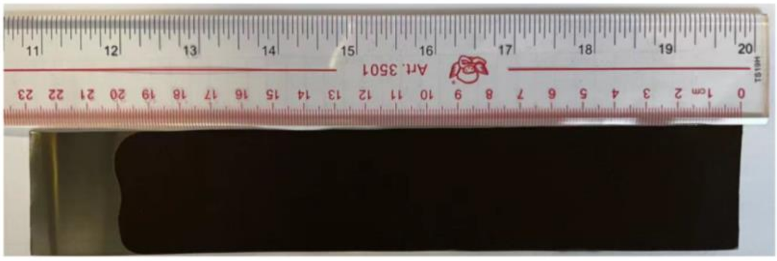

Supplementary Fig. 39 Digital image of NVO electrode for AA-Zn/NVO cell.

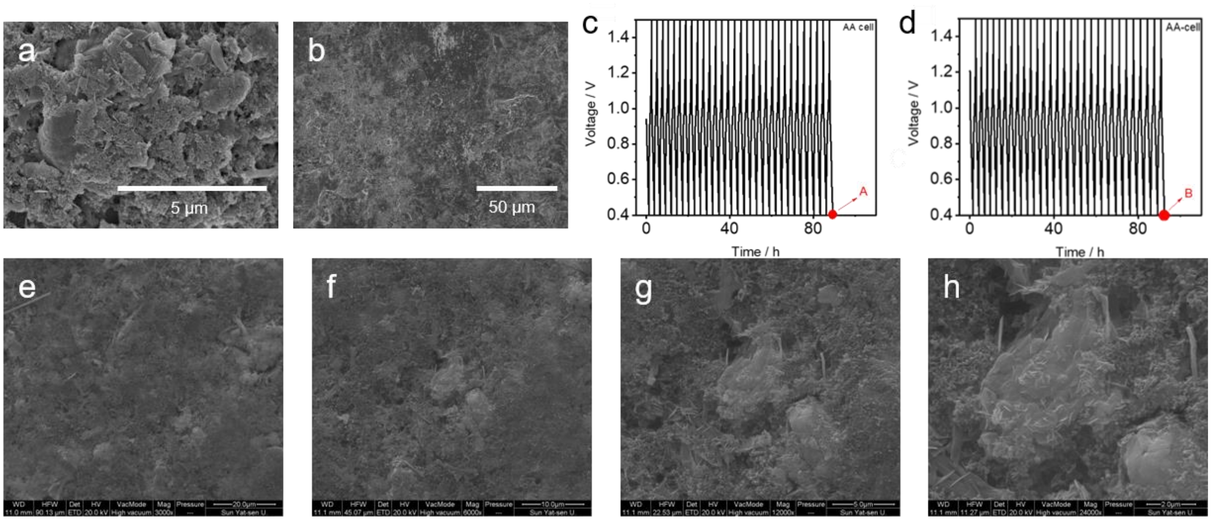

Supplementary Fig. 40 SEM images of **a** NVO cathode and **b** Zn anode of AA-Zn/NVO with WSE after cycling stability test. **c** GCD curves of associated electrochemical data for Supplementary Fig. 40a. The tested voltage window for AA-Zn/NVO with WSE is 0.4-1.5 V. The current density is 0.66 C-rate. The SEM image in Supplementary Fig. 40a was used to investigate the morphology change of NVO after cycling test in AA-Zn/NVO with WSE (discharged state), which was recorded at point A in Supplementary Fig. 40c. **d** GCD curves of newly assembled AA-Zn/NVO with WSE for reproducibility purposes. **e-h** SEM images from different magnifications of NVO electrode tested in newly assembled AA-Zn/NVO with WSE (discharged state, recorded at point B in Supplementary Fig. 40d). The SEM images of NVO electrode tested in AA-Zn/NVO with WSE indicates that the morphology of NVO at low magnification is relatively homogeneous, while some clumps are observed at high magnification, which is also similar to the SEM images of Supplementary Fig. 40a.

Supplementary Table S1. ε and DN of some common organic solvent**.**

Solvent	ε	DN
Cyclohexane	2.0	0
1,4-Dioxane	2.2	14.8
Ethyl acetate	6.0	17.1
Butanone	18.5	17.4
Acetone	20.7	17.0
Acetonitrile	37.5	14.1
Water	80.4	18

Supplementary Table S2. Comparison of the electrochemical stability of state-of-the-art Zn metal electrodes with different electrolyte formula.

Electrolyte	Test condition	Depth of discharge	Lifespan	Ref.
0.5 M $ZnSO_4$-H_2O-C_4H_8O	1 mA cm^{-2}, 1 mAh cm^{-2}	1.7%	2200 h	This work
	60 mA cm^{-2}, 60 mAh cm^{-2}	88.2%	70 h	
1 M $Zn(TFSI)_2$+ 20 M LiTFSI-H_2O	0.2 mA cm^{-2}	—	170 h	1
4 M 1-ethyl-3-methylimidazo-lium + 1 M $ZnSO_4$	1 mA cm^{-2}, 1 mAh cm^{-2}	—	500 h	2
2 M ZnSO4-H_2O-NMP	1 mA cm^{-2}, 1 mAh cm^{-2}	—	540 h	3
3 M $ZnSO_4$-H_2O-EG	0.5 mA cm^{-2}, 0.5 mAh cm^{-2}	—	~1380 h	4

2 M $Zn(OTF)_2$-H_2O-DMC	1 mA cm^{-2}, 0.5 mAh cm^{-2}	1.7%	~1100 h	5
1 M $ZnSO_4$ + 0.5 M Na_2SO_4 + PAM-H2O	1 mA cm^{-2}, 1 mAh cm^{-2}	—	180 h	6
1 M ZnSO4-H2O + glucose	1 mA cm^{-2}, 1 mAh cm^{-2}	—	2000 h	7
1.3 m $ZnCl_2/H_2O$−DMSO	0.05 mA cm^{-2}, 0.5 mAh cm^{-2}	—	1000 h	8
$Zn(ClO_4)_2 \cdot 6H_2O$+ succinonitrile	0.05 mA cm^{-2}, 0.5 mAh cm^{-2}	—	800 h	9
$Zn(TFSI)_2$-Ace	0.1 mA cm^{-2}, 0.05 mAh cm^{-2}	—	500 h	10

Supplementary Table S3. Comparison of the electrochemical stability of Zn-V battery with different electrolyte chemistry.

Cathode	Anode	Electrolyte	Capacity retention	Ref.
NVO	Zn foil	0.5 M $ZnSO_4$ + 12.5 v/v% butanone	99.1% after 20000 cycles at 5 A g^{-1}	This work
V_2O_5/graphene oxide	Zn foil	21 m LiTFSI + 3 m $ZnOTf_2$+10 wt % PVA	93% over 600 cycles at 500 mA g^{-1}	11
V_2O_5	Zn foil	2 M $ZnSO_4$ + 25% sulfolane	70% after 500 cycles at 10 A g^{-1}	12
V_2O_5	Zn foil	$Zn(OTf)_2$ + 30% 2-propanol	79.6%% after 1500 cycles at 2 A g^{-1}	13
$V_2O_5 \cdot nH_2O$	Zn foil	2 M $Zn(OTf)_2$ + 40% DME	93.1% after 2000 cycles at 2 A g^{-1}	14
NVO	Zn foil	2 M $ZnSO_4$ + 0.1 M ImS	88% after 3000 cycles at 20 A g^{-1}	15
$Zn_xV_2O_5 \cdot nH_2O$	Zn foil	3 M $ZnSO_4$ + 68% EG	89.6% after 500 cycles at 500 mA g^{-1}	4

Supplementary Table S4. The cell parameters for laboratory-scale coin cell in the literature and AA-Zn/NVO cell in this work.

	Thickness of Zn anode (mm)	N/P ratio	E/C ratio (g Ah^{-1})	Total discharge capacity (mAh)
Coin cell	0.1	~84:1	~92	~1
AA cell (This work)	0.03	19:1	19.6	101.7

Supplementary Reference

1. Wang, F. *et al.* Highly reversible zinc metal anode for aqueous batteries. *Nat. Mater.* **17**, 543–549 (2018).
2. Zhang, Q. *et al.* Designing Anion-Type Water-Free Zn^{2+} Solvation Structure for Robust Zn Metal Anode. *Angew. Chem. Int. Ed.* **133**, 23545–23552 (2021).
3. Li, T. C. *et al.* A Universal Additive Strategy to Reshape Electrolyte Solvation Structure toward Reversible Zn Storage. *Adv. Energy Mater.* **12**, 2103231 (2022).
4. Qin, R. *et al.* Tuning Zn^{2+} coordination environment to suppress dendrite formation for high-performance Zn-ion batteries. *Nano Energy* **80**, 105478 (2021).
5. Dong, Y. *et al.* Non-concentrated aqueous electrolytes with organic solvent additives for stable zinc batteries. *Chem. Sci.* **12**, 5843–5852 (2021).
6. Zhang, Q. *et al.* The Three-Dimensional Dendrite-Free Zinc Anode on a Copper Mesh with a Zinc-Oriented Polyacrylamide Electrolyte Additive. *Angew. Chem. Int. Ed.* **58**, 15841–15847 (2019).
7. Sun, P. *et al.* Simultaneous Regulation on Solvation Shell and Electrode Interface for Dendrite-Free Zn Ion Batteries Achieved by a Low-Cost Glucose Additive. *Angew. Chem. Int. Ed.* **60**, 18247–18255 (2021).
8. Cao, L. *et al.* Solvation Structure Design for Aqueous Zn Metal Batteries. *J. Am. Chem. Soc.* **142**, 21404–21409 (2020).
9. Yang, W. *et al.* Hydrated Eutectic Electrolytes with Ligand-Oriented Solvation Shells for Long-Cycling Zinc-Organic Batteries. *Joule* **4**, 1557–1574 (2020).
10. Qiu, H. *et al.* Zinc anode-compatible in-situ solid electrolyte interphase via cation solvation modulation. *Nat. Commun.* **10**, 5374 (2019).
11. Zhang, H., Liu, X., Li, H., Qin, B. & Passerini, S. High-Voltage Operation of a V_2O_5 Cathode in a Concentrated Gel Polymer Electrolyte for High-Energy Aqueous Zinc Batteries. *ACS Appl. Mater. Interfaces* **12**, 15305–15312 (2020).
12. Wang, M. *et al.* High-Capacity Zinc Anode with 96% Utilization Rate Enabled by Solvation Structure Design. *Angew. Chem. Int. Ed.* **62**, (2023).

13. Ma, Q. *et al.* Regulation of Outer Solvation Shell Toward Superior Low-Temperature Aqueous Zinc-Ion Batteries. *Adv. Mater.* **34**, 2207344 (2022).
14. Ma, G. *et al.* Reshaping the electrolyte structure and interface chemistry for stable aqueous zinc batteries. *Energy Storage Mater.* **47**, 203–210 (2022).
15. Lv, Y. *et al.* Engineering a self-adaptive electric double layer on both electrodes for high-performance zinc metal batteries. *Energy Environ. Sci.* **15**, 4748–4760 (2022).
