# Supplementary material for: A weakly solvating electrolyte towards practical rechargeable aqueous zinc-ion batteries
Source: Nat Commun. 2024 Jan 5;15:302. doi: 10.1038/s41467-023-44615-y (PMC10770389; doi:10.1038/s41467-023-44615-y)
Supplement: Supplementary file 3 — Description of Additional Supplementary Files [file 41467_2023_44615_MOESM3_ESM.pdf]

Description of Additional Supplementary Files

Supplementary Movie 1. The integrated AA-Zn/NVO with WSE can provide sufficient power to drive a remote-controlled car.
